# Cases

**Published:** 1875-07

**Authors:** R. L. Rea

**Affiliations:** Chicago


Article VI.

### CASES.

By R. L. REA, M.D., Chicago.

Mrs. M——, aged ——, very corpulent; suffered with double hernia sixteen years ago. They were frequently reduced. Trusses worn. The left side was finally cured, but the right at last became irreducible, and remained so for several years, and gave no trouble.

On the night of March 8th, she was taken suddenly with a pain about the region of the rupture, and extending over the abdomen, which she supposed resulted from unusual diet she had taken for supper. Dr. Tomboecken, her family physician, was called, and detecting the difficulty, made faithful efforts at reduction, without success, the symptoms gradually passing into the well-defined ones of strangulation. I was called in consultation with Dr. T. on the ninth, when the Doctor's fruitless efforts were repeated, varied by position, etc.

Satisfied that further attemps were worse than wasted time, at the Doctor's request I operated.

The tumor, pyriform, about the size of an ordinary fist, lay obliquely from above, down and inwards, the narrow end terminating at the position of the internal abdominal ring, and the large end lying over the situation of the external ring, the whole in position over the inguinal canal. I made my incisions in the usual manner for inguinal hernia, and opening the sac, passed my finger toward the internal abdominal ring—the supposed seat of the stricture—to find it end in a cul de sac. Reversing the direction of my finger for a direct inguinal hernia, I found a similar condition. It then seemed certain that the kind of hernia had been mistaken, which exploration confirmed. That it was a femoral hernia, which, escaping as usual, had, after turning upward after its escape through the saphenous opening, as it further developed in size, ascended and arranged itself in the manner I have described. The hernia was incarcerated, and though I relieved the stricture, the lighted inflammation proved fatal.

The peculiarity and interest of the case centre in the fact of the perfect counterfeit of one form of hernia by another—in this case of little practical use, but in others, capable of reduction, of inestimable value.

The great obesity of the patient added much to the embarrassment of the diagnosis.

Maggie S——, Irish servant, aged 20; light complexion, medium sized; always healthy, without any peculiarity perceptible in her organization; has menstruated regularly since she was fourteen. She began to menstruate December 28th, and on Monday morning, the 30th, went through a rain storm some distance from a relative's, where she had stopped over night, to her place, getting her feet and clothing thoroughly wet. Her menses ceased, and she felt badly during the day. She consulted me at my office, on Thursday, Jan. 2, for a "bleeding from her chest," as she expressed it, and supposing her diagnosis of hemoptysis correct, I had her remove her clothing in order to examine her lungs, when the nature of her bleeding from her chest was manifest as vicarious menstruation from the mammary glands; her chemise over both breasts being thoroughly stained, amounting, as I should judge, to two ounces. She stated that, ceasing on Monday, the menses appeared on Tuesday from the nipples, continuing there Wednesday, and resuming their normal direction on Thursday and ceasing Friday as they did, with no seeming interference with health or leaving any symptoms requiring treatment. She was alarmed at the mammary discharge, under the impression that there was hæmorrhage of the lungs, and hence consulted me. The discharge had dried on her clothing, and hence I cannot give an opinion as to its quality.

The subsequent month she menstruated regularly and naturally, but in February had a return of the December abnormity, which passed over in the same way and without treatment.

